# Determining who responds better to a computer- vs. human-delivered physical activity intervention: results from the community health advice by telephone (CHAT) trial

**DOI:** 10.1186/1479-5868-10-109

**Published:** 2013-09-22

**Authors:** Eric B Hekler, Matthew P Buman, Jennifer Otten, Cynthia M Castro, Lauren Grieco, Bess Marcus, Robert H Friedman, Melissa A Napolitano, Abby C King

**Affiliations:** 1 School of Nutrition and Health Promotion, Arizona State University, 500 N. 3rd St, Phoenix, AZ 85004, USA; 2 School of Medicine, Stanford University, Stanford, USA; 3 Department of Family and Preventive Medicine, University of California, San Diego, CA, USA; 4 School of Medicine, Boston University and Boston Medical Center, Boston, USA; 5 George Washington University, Washington, DC, USA

**Keywords:** Physical activity, Intervention, Moderation, Interactive voice response, Behavioral intervention technology system, Targeting

## Abstract

**Background:**

Little research has explored who responds better to an automated vs. human advisor for health behaviors in general, and for physical activity (PA) promotion in particular. The purpose of this study was to explore baseline factors (i.e., demographics, motivation, interpersonal style, and external resources) that moderate intervention efficacy delivered by either a human or automated advisor.

**Methods:**

Data were from the CHAT Trial, a 12-month randomized controlled trial to increase PA among underactive older adults (full trial N = 218) via a human advisor or automated interactive voice response advisor. Trial results indicated significant increases in PA in both interventions by 12 months that were maintained at 18-months. Regression was used to explore moderation of the two interventions.

**Results:**

Results indicated amotivation (i.e., lack of intent in PA) moderated 12-month PA (*d* = 0.55, *p* < 0.01) and private self-consciousness (i.e., tendency to attune to one’s own inner thoughts and emotions) moderated 18-month PA (*d* = 0.34*, p* < 0.05) but a variety of other factors (e.g., demographics) did not (*p* > 0.12*)*.

**Conclusions:**

Results provide preliminary evidence for generating hypotheses about pathways for supporting later clinical decision-making with regard to the use of either human- vs. computer-delivered interventions for PA promotion.

## 

Regular physical activity is a key factor in the prevention of a variety of chronic diseases (e.g., atherosclerotic heart disease, hypertension, diabetes, obesity), yet the majority of middle-aged and older adults remain insufficiently active [1]. Cost-effective and efficient methods for promoting physical activity that have greater public health impact are needed [2,3]. Exploring moderators of intervention success can improve the efficacy of health behavior interventions by identifying “…for whom or under what conditions the treatment works” [p 878 [4]]. Identifying subgroups who respond differently to different types of interventions (e.g., based on intervention content or means of delivery) is a key priority in the physical activity promotion field as it fosters development of more focused and targeted interventions [5-9] that, by extension, should ultimately lead to more cost-effective and efficient strategies for physical activity promotion [9]. It ought to be possible to target specific interventions based on a variety of personal, social, and environmental factors that can be assessed prior to starting a trial.

A rapidly growing body of research is exploring the use of various technologies such as interactive voice response systems, text messaging, web pages, and smartphones for promoting physical activity and other health behaviors [10-14]. Much of this previous research has largely focused on determining if behavior change technologies can effectively promote physical activity. As there is increasing evidence that they can promote physical activity at comparable levels to human counseling [10,15], an important follow-up is to identify which intervention works better for whom. Specifically, if two interventions are found to be equally effective, other factors that determine responsivity or success become important for supporting clinical decision making.

The Community Health Advice by Telephone (CHAT) trial tested the efficacy of 12-month human vs. automated computer advisors that delivered physical activity interventions relative to a time-matched health education control [10,15]. In the CHAT trial, the content, timing, and delivery channel (i.e., both via the telephone) of the interventions were identical, with only the type of advisor (i.e., human vs. automated) differing between the two groups. Study results showed that both intervention strategies promoted significant increases in moderate-to-vigorous intensity physical activity (Mod+) relative to the health-education control over the course of 12 months, with mean minutes of Mod + for both intervention arms above the national guidelines of 150 minutes per week [16]. Additional results revealed that both arms maintained physical activity gains at 18 months with only minimal, self-initiated contact with their assigned human or automated advisor [17].

Since results indicate that both the human and automated advisors effectively promoted physical activity initiation and maintenance, a secondary aim of the CHAT trial was to evaluate potential moderators of the intervention-physical activity relationship to provide preliminary empirical evidence to support later clinical decision making for determining which intervention is “right” for whom [p 102, [15]]. Based on this, a number of psychosocial variables were measured at baseline within the CHAT trial to explore who responds best to a human vs. technology delivered intervention. Overall, the data from the CHAT trial is an excellent dataset for generating hypotheses about informing later clinical decisions about which intervention to recommend to whom as both interventions share the same content, amount of contact, delivery channel, both were optimized for their own delivery modality (e.g., while humans followed a script, they were still allowed to personalize the advice) and both were found to be similarly efficacious at promoting 12-month physical activity increases and 18-month maintenance of physical activity [17].

Few if any studies have explored who may respond better to a technology-based intervention compared to a human delivered intervention for physical activity initiation and maintenance. As discussed by King et al. [9], a variety of domains could conceivably be used to target an intervention such as demographic or psychosocial factors. In addition, there is a growing body of research emphasizing the importance of not just initiation of a health behavior but maintenance as well [18], and that baseline factors that are important in maintenance compared to initiation may be different [9,19]. As there is little to no evidence exploring key target variables for predicting differential intervention responses to technology vs. human interventions for physical activity initiation or maintenance, we chose to focus on four broad domains (i.e., demographics, motivation, interpersonal style, and social resources) based on more general research on targeting [9,19] and *a priori* expectations [15]. We chose these four broad domains largely based on known differences in communication style that were present between our human vs. automated advisor interventions. Specifically, while the automated advisor provided the same intervention content (e.g., goal setting), it could not provide the full realm of interactions that are possible between humans. For example, even though the human advisor followed a structured script and protocol, the human advisors could still provide subtle shifts in tone or slightly rephrased advice that could be more in line with the current needs of a person compared to an automated advisor. Alternatively, there might be conditions in which the automated advisor’s utilitarian focus on physical activity promotion without any other detours in conversation could actually be beneficial for certain individuals.

With regard to demographics, we chose to specifically explore if age, gender, ethnicity, or marital status might impact differential responses. Among these demographics, we believed gender would be the most important as we hypothesized that women may implicitly respond better to a human intervention whereas men may respond better to an automated advisor. We also believed that age might play a role as those who are older may have had a harder time working with the automated advisor and thus not respond as well. That said, we were also working in a restricted age range population (i.e., already older adult group) and thus had a restricted range to explore this moderation effect. We included the other demographics to foster hypothesis generation and because if they were significant moderators, they could easily be utilized for targeting in clinical research.

With regard to motivation, self-determination theory [20] was the guiding theoretical model for the selected measures. We hypothesized that intrinsic motivation/autonomy may differentially impact responsiveness to a human vs. automated advisor (e.g., individuals with lower autonomy/intrinsic motivation at baseline would require a human advisor). We hypothesized this because we believed it possible that the human advisors, even after closely following the protocol, might have been able to expand their repertoire of discussion points to coax less motivated individuals to action better than an automated advisor. With regard to interpersonal style, we postulated that persons who experienced social anxiety would fare better with the automated advisor as socially anxious people feel discomfort in the presence of others and avoid those interactions, but an automated advisor may not trigger this experience. Finally, we focused on baseline social support because it may function as a “buffer” for reduced social contact or connectedness when counseling is delivered through an automated advisor. It is important to emphasize, however, that these hypotheses were based largely on our interpretation of behavioral theory and not previous empirical data. As such, we see this work as primarily hypothesis generation rather than confirmatory hypothesis testing.

## Methods

### Study design

The methods and the results, including descriptions reported to conform to the CONSORT guidelines for clinical trials, for the Community Health Advice by Telephone (CHAT) trial have been described in detail elsewhere [10,15-17]. In brief, the CHAT trial evaluated the 12-month effects, relative to a control arm, of two telephone-delivered physical activity guidance and support interventions. Following completion of this trial phase, participants in the two intervention arms were followed for an additional 6-month period with only self-initiated contact with their respective advisors (i.e., either the human or automated advisor). Study results revealed significant improvements in Mod + for both study arms over 12 months [16] that were maintained at 18 months [17]. Blinded evaluation occurred at baseline, 6, and 12 months and an additional assessment was conducted at 18 months for the two intervention arms only. The Stanford Office of Human Subjects Research approved the study protocol.

#### Interventions

The counseling content delivered in both intervention arms was based on Social Cognitive Theory and the Transtheoretical Model [10,15]. Individuals randomized to the automated advice arm received the same intervention content, schedule of contact, number of contacts, and time per contact as the human advice arm. Full details, including descriptive statistics on appropriate fidelity checks to ensure similar content between the two groups have been reported previously [10,15,17] and will be described briefly here. Specifically, both the human advisor and automated advisor were scheduled to contact the participants 15 times during the intervention year. After the 12 month period, participants were given the option to call their advisors as they saw fit. As reported previously [17], significantly more individuals called in to the Human arm during the Maintenance phase (31.9% [n = 23] never called in) compared with the automated arm (58.1% [n = 43] never called in; *t* = 3.4, p < .001).

The following content was delivered by both the human and automated advisor: physical activity assessment, progress evaluation, individualized problem-solving, goal-setting, feedback, and delivery of positive support and tailored advice based on an individual’s stage of change. Further, the human advisors were given scripts to follow that were parallel to the automated advisor scripts. The human advisor scripts provided specific content areas to cover and suggested language to use to cover each content area that was identical to the automated advisor. The scripts for the human advisors mimicked the logic used for determining the appropriate content to be delivered to the participant via the automated advisor. For example, the script for the human advisor included strategies for assessing a participant’s current stage of change (e.g., contemplation, preparation, action). After this determination, specific stage-tailored scripts were used to provide the content for both the human and automated advisor. The users of the automated advisor communicated using the touch-tone keypad of their telephones [21,22], whereas the human advisors engaged in live verbal conversation. Since the human advisors used live verbal communication, some deviation from the scripts was allowed as already mentioned. All human counselors were women with at least a bachelor’s degree and were supervised by CMC. Similarly, the automated advisor was a pre-recorded women’s voice of a professional “voice talent” who read aloud all of the scripts. The automated advisor “spoke” to participants over the telephone using computer-controlled “sound bites” of the prerecorded statements and questions.

A variety of fidelity checks were accomplished throughout the study to further ensure the interventions were consistent [10,15]. Specifically, all human counseling sessions were recorded and regularly evaluated via an independent review of counselor summary forms at the time of each contact and independent review of audio-taped telephone contacts. Approximately one third of total telephone contacts, selected at random, were reviewed by other counseling personnel for independent coding and verification that the counselors were not straying from the script and content. Fidelity checks for the automated advisor included semiweekly evaluation of the technical performance reports generated by the system (e.g., information about call rates, length of calls, etc) and also daily monitoring of the automated system’s “help line,” which was used to allow participants to report any problems with the automated advisor. Descriptive statistics on the fidelity between the automated advisor and human advisor were reported previously [10]. Briefly, the two groups had similar number of completed calls (i.e., *M*_*humanadvisor*_ *= 13.1 ± 2.5* vs. *M*_*automatedadvisor*_ *= 11.8 ± 4.8 calls completed*), the human advisors did have a significantly longer call length compared to the automated advisor (i.e., *M*_*humanadvisor*_ *= 10.7 ± 5.0* vs. *M*_*automatedadvisor*_ *= 6.6 ± 2.2 min/call).* Overall fidelity checks on the quality of the content suggested good fidelity to the intervention content and script between the human advisor and automated advisor.

### Participants

Eligibility criteria for the study were: ages 55 years and older; physically underactive (i.e., not engaged in more than 60 min/week of Mod + over the previous 6 months), free of any medical condition that would limit participation in exercise, and able to speak and understand English sufficiently to provide informed consent and participate in study intervention and assessment procedures. Eligible individuals were randomized to a 12-month home-based moderate-intensity physical activity (primarily walking) adoption program delivered via a trained telephone counselor (Human Advice arm, n = 73), a similar program delivered via an automated, computer-controlled interactive telephone system (Automated Advice arm, n = 75), or a general health education control arm (n = 70). For these analyses, we only focused on the human and automated advice arms.

### Measurement of physical activity

#### Stanford 7-day physical activity recall (PAR)

The 7-Day PAR [23] was the primary measure of physical activity. The PAR is a well validated structured interview that asks participants to estimate their total time during the past seven days engaging in four physical activity intensity categories: sleep, moderate, hard, and very hard physical activity [24]. The PAR has been shown to have good reliability [25], and concurrent validity [26] at these intensities. The primary outcomes for these analyses were 12-month and 18-month PAR estimates of energy expenditure from Mod + (in kcal/kg/day).

### Measurement of proposed moderator variables

Proposed moderators included demographics (i.e., age, gender, ethnicity, and marital status) and physical activity-specific measures of motivation based on self-determination theory (e.g., amotivation, autonomous motivation), interpersonal style (i.e., social anxiety and self-consciousness), and social support. These variables were assessed at baseline prior to study arm random assignment.

Self-determination theory constructs [20] were measured using the Treatment Self-Regulation Questionnaire for Exercise (TSRQ-E) [27]. This measure includes the following subscales: autonomous motivation (e.g., “I would exercise regularly because I feel that I want to take responsibility for my own health,” current sample *α* = 0.87), introjected regulation (e.g., “I would exercise regularly because I would feel guilty or ashamed of myself if I did not exercise regularly,” current sample *α* = 0.83), external regulation (e.g., “I would exercise regularly because I feel pressure from others to do so,” current sample *α* = 0.72), and amotivation (e.g., “I really don’t think about it,” current sample *α* = 0.49). Internal consistency of the items was very good across a large sample (N > 2000, *α’*s > 0.73) and the measures evidenced good validity [27].

Self consciousness and social anxiety were measured using the self-consciousness survey-revised (SCS-R [28]). The self-consciousness survey-revised is a validated measure that includes subscales on private self-consciousness (e.g., “I am always trying to figure myself out,” current sample *α* = 0.76), public self-consciousness (e.g., “I usually worry about making a good impression,” current sample *α* = 0.80), and social anxiety (e.g., “It takes me time to get over my shyness in new situations,” current sample *α* = 0.75). Previous research indicates that it is a reliable and valid scale [28].

Social support was assessed using the social support for exercise (SSE) scale [29]. The SSE is a well-validated and widely used scale that measures social support for being physically active. It has been used across a wide variety of demographic groups including older adults [30]. It includes subscales on the supportiveness of both family (current sample, *α* = 0.92) and friends (current sample, *α* = 0.87).

### Statistical analyses

Linear regression was used to conduct these analyses [31]. As the primary questions focused on differences between the two active interventions, the attention-control arm was not included in any analyses. All primary moderator variables of interest (i.e., demographics, subscales from the TSRQ-E, SCS-R, SSE, and study arm assignment) were centered and then an interaction term was calculated between the moderator variable and study arm assignment [32]. The primary outcome variables were 12-month and 18-month average daily PAR-based Mod + energy expenditure values. For 12-month outcome models, baseline PAR values were included in the models as a covariate. For 18-month analyses, a residualized change score, which represented the unique variance of change in physical activity from baseline to 12 months [33], was entered into the model as a covariate to control for any changes that occurred during the intervention. We incorporated intent-to-treat principles for the primary outcomes of interest, whereby, for participants with missing or incomplete PAR data at 12 months (n = 21) and 18 months (n = 33; 18 in Human arm, 15 in Automated arm, between-arm difference *p* > 0.10), their baseline values were used. For some participants missing 12-month data (n = 8), secondary physical activity data sources were available (i.e., 12-month CHAMPS questionnaires, logged reports of physical activity occurring during the 7-12 month intervention period that were collected as part of all exercise advisor [automated or human] interactions). If the data from these secondary sources indicated that participants were at least as active as their 6-month PAR data indicated, then the 6-month values were used [see: 10, [34] for a full discussion on this imputation method]. The intent-to-treat analyses are reported for the PAR 12-month and 18-month outcomes. Alpha was set at .05 using a two-tailed test of significance for all interactions.

## Results

### Descriptive statistics

Table 1 summarizes demographic characteristics, other baseline moderator variables, and 12-month and 18-month physical activity variables. Participants (n = 148) were mostly Caucasian, women, overweight, and had a mean age of 60.7 ± 5.9 years. There were no significant differences between the two arms at baseline on any demographic variables. Participants in the human advisor arm, on average, reported greater private self-consciousness at baseline relative to the automated advisor arm (*p* < 0.05). There were no other significant differences between the human and automated advisor arms at baseline (see Table 1).

**Table 1:** Demographics, baseline moderators, and 12 m physical activity

**N**	**Automated**		**Human**			**All**	
	**75**		**73**			**148**	
**Demographics**					*p*		
White/Caucasian, n (%)	67 (90.5%)		59 (80.8%)		0.09	126 (85.7%)	
Women, n (%)	49 (65.3%)		50 (68.5%)		0.68	99 (66.9%)	
Married/Co-habituating, n (%)	52 (70.3%)		49 (67.1%)		0.63	101 (68.7%)	
drop-out, n (%)	6 (8.0%)		5 (6.9%)		0.79	11 (7.4%)	
	M ± SD	Min-Max	M ± SD	Min-Max	*p*	M ± SD	Min-Max
Years In School	16.4 ± 1.8	12.0-18.0	16.2 ± 1.9	12.0-18.0	0.63	16.3 ± 1.8	12.0-18.0
Age	61.1 ± 5.6	55.0-77.0	60.4 ± 6.3	52.0-78.0	0.49	60.7 ± 5.9	52.0-78.0
BMI	29.2 ± 5.1	17.9-39.3	29.9 ± 5.2	19.1-42.3	0.40	29.5 ± 5.2	17.9-42.3
Baseline Mod + (kcal/kg/day)	0.7 ± 1.1	0.0-6.3	0.8 ± 1.0	0.0-4.1	0.67	0.8 ± 1.0	0.0-6.3
**Baseline Moderators**							
Amotivation for exercise (1-7)	2.0 ± 1.0	1.0-7.0	2.1 ± 1.1	1.0-6.0	0.67	2.0 ± 1.0	1.0-7.0
Autonomy for exercise (1-7)	6.1 ± 0.9	3.7-7.0	6.2 ± 0.9	3.2-7.0	0.79	6.2 ± 0.9	3.2-7.0
Introjected motivation for exercise (1-7)	3.5 ± 1.7	1.0-7.0	3.6 ± 1.5	1.0-7.0	0.79	3.6 ± 1.6	1.0-7.0
External motivation for exercise (1-7)	2.3 ± 1.2	1.0-6.3	2.5 ± 1.1	1.0-5.5	0.21	2.4 ± 1.2	1.0-6.3
Family social support for exercise (0-75)	34.7 ± 13.3	0.0-75.0	32.6 ± 15.4	0.0-73.0	0.39	33.7 ± 14.4	0.0-75.0
Friend Social Support for Exercise (0-75)	29.0 ± 10.1	0.0-57.7	29.0 ± 9.0	0.0-57.0	1.00	29.0 ± 9.5	0.0-57.7
Social anxiety (0-18)	6.1 ± 4.1	0.0-17.0	7.4 ± 4.3	0.0-17.0	0.07	6.7 ± 4.2	0.0-17.0
Private self-consciousness (0-26)	12.9 ± 4.7	0.0-23.0	14.5 ± 5.5	0.0-26.0	0.07	13.7 ± 5.2	0.0-26.0
Public self-consciousness (0-20)	10.3 ± 4.6	0.0-20.0	11.5 ± 5.5	0.0-20.0	0.10	10.9 ± 4.7	0.0-20.0
12 m PAR Mod + (kcal/kg/day)	1.5 ± 1.5	0.0-7.6	1.6 ± 1.2	0.0-5.6	0.93	1.6 ± 1.4	0.0-7.6
18 m PAR Mod + (kcal/kg/day)	1.4 ± 1.6	0.0-9.6	1.5 ± 1.4	0.0-5.6	0.14	1.5 ± 1.5	0.0-9.6

*p* values represent *t*-test or Chi-square analyses comparing automated vs. human study arms; *Mod +* moderate- to vigorous-intensity physical activity.

### Moderator analyses

**Figure 1:**
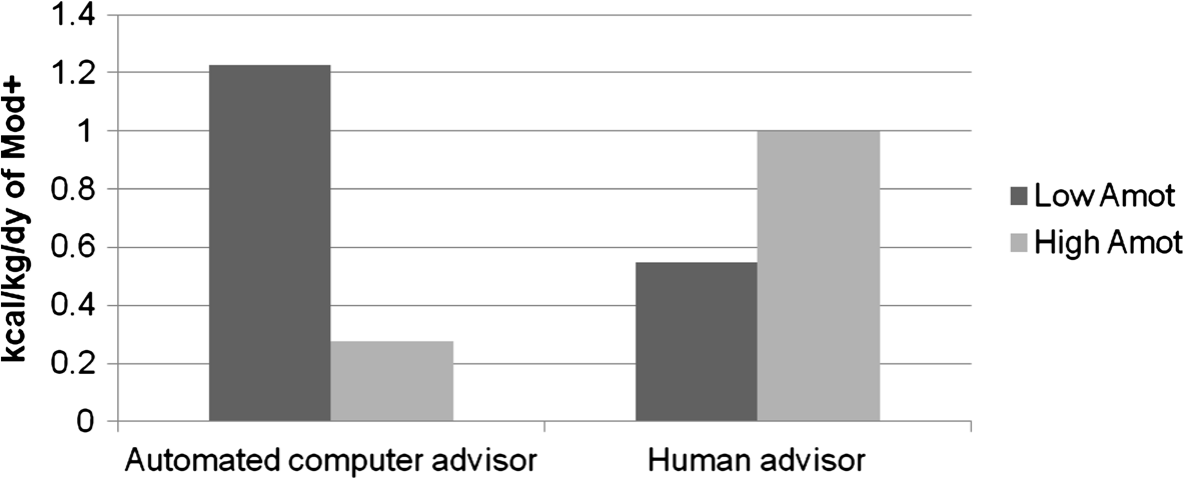
**Amotivation moderating 12 month physical activity.** Note: Results above are predicted values based on a multiple regression model that included arm (t = 0.11, *df* = 147, *p* = 0.91), amotivation (*t* = -1.2, *df* = 147, *p* = 0.24), baseline moderate- to vigorous-intensity physical activity (Mod+) (*t* = -7.0, *df* = 147, *p* < 0.0001), and arm X amotivation (*t* = 3.31, *df* = 147, *p* = 0.0012). Using standard conventions for modeling interaction effects, the y-axis represents the predicted value at 12 months of physical activity (i.e., kilocalories burned per kilogram per day that was in the moderate to vigorous intensity range) for those high and low in amotivation between the automated advisor and human advisor groups. Low Amot = One Standard deviation below mean for amotivation; High Amot = One standard deviation above the mean for amotivation.

At 12 months, results indicated that baseline amotivation moderated intervention effects (*d* = 0.55, *β* = 0.27, *p* = 0.006). Examination of the plot (see Figure 1) suggests that participants reporting more baseline amotivation (i.e., lack of intent or interest in being physically active) to exercise responded better to a human, whereas those who reported less baseline amotivation (i.e., more initial physical activity interest) responded better to automated advice. No other variable significantly moderated 12-month physical activity levels (*ps* > 0.12).

**Figure 2:**
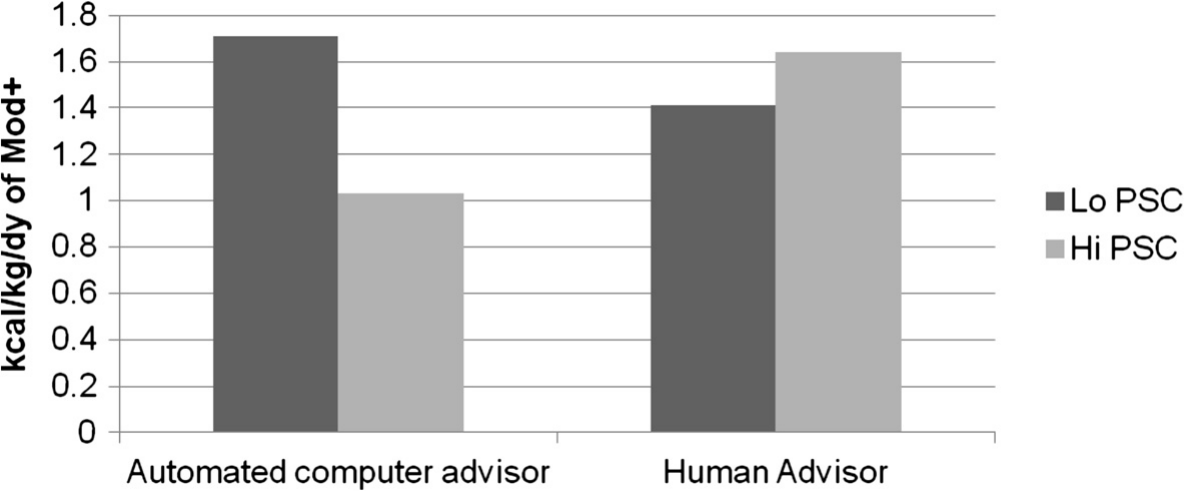
**Private self-consciousness moderating 18 month physical activity.** Note: Results above are predicted values based on a multiple regression model that included Arm (t = 0.72, *df* = 147, *p* = 0.47), private self-consciousness (*t* = -1.02, *df* = 147, *p* = 0.31), residualized change score of physical activity from baseline to 12 months (*t* = 6.25, *df* = 147, *p* < 0.0001), and arm X private self-conscious (*t* = 2.04, *df* = 147, *p* < 0.05) predicting moderate- to vigorous-intensity physical activity (Mod+) at 18 months. Using standard conventions for modeling interaction effects, the y-axis represents the predicted value at 18 months of physical activity (i.e., kilocalories burned per kilogram per day that was in the moderate to vigorous intensity range) for those high and low in private self-consciousness between the automated advisor and human advisor groups. Low PSC = one Standard deviation below mean for private self-consciousness; High PSC = One standard deviation above the mean for private self-consciousness. This is based on previous conventions for graphing moderation analyses as described above.

**Figure 3:**
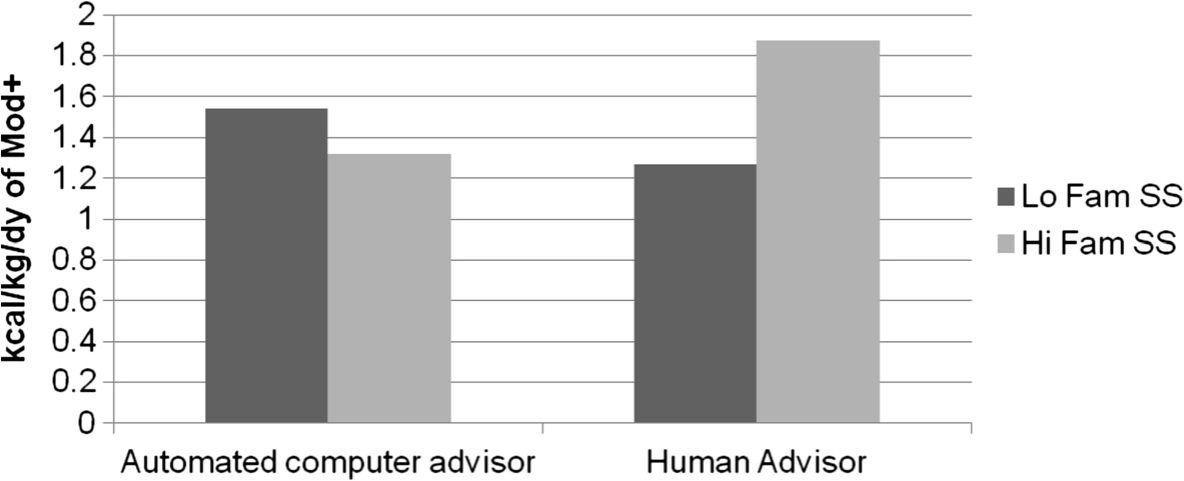
**Trend result of family social support moderating 18 month physical activity.** Note: Results above are predicted values based on a multiple regression model that included Arm (t = 0.65, *df* = 147, *p* = 0.52), family social support (*t* = 0.86, *df* = 147, *p* = 0.39), residualized change score of physical activity from baseline to 12 months (*t* = 6.54, *df* = 147, *p* < 0.0001), and arm X family social support (*t* = 1.88, *df* = 147, *p* = 0.06) predicting moderate- to vigorous-intensity physical activity (Mod+) at 18 months. Using standard conventions for modeling interaction effects, the y-axis represents the predicted value at 18 months of physical activity (i.e., kilocalories burned per kilogram per day that was in the moderate to vigorous intensity range) for those high and low in social support at baseline between the automated advisor and human advisor groups. Low SS = one Standard deviation below mean for family social support; High SS = One standard deviation above the mean for family social support. This is based on previous conventions for graphing moderation analyses as described above.

At 18 months, analyses revealed that private self-consciousness significantly moderated 18-month maintenance of physical activity (*d* = 0.34, *β* = 0.15, *p* = 0.04). Specifically, those who were high in private self-consciousness and randomized to the automated advisor did not maintain their physical activity levels as well as all other types of individuals (i.e., those low in private self-consciousness in the automated advisor arm and all individuals randomized to the human advisor arm, see Figure 2). Although not statistically significant, results also suggested a trend for baseline family social support moderating maintenance of physical activity at 18 months (*d* = 0.31, *β* = 0.13, *p* = 0.07). Specifically, those individuals who were high in family social support and who had been randomized to the human advisor maintained their physical activity levels better than all others (see Figure 3).

## Discussion

These secondary analyses indicated two moderators of physical activity based on intervention delivery method, with different moderator results at 12 and 18 months. At 12 months, individuals with high amotivation (i.e., less interested in physical activity at baseline) engaged in more physical activity at 12 months when assigned to a human advisor whereas those with low amotivation (more initial interest in becoming more active) responded better to an automated advisor. At 18 months, private self-consciousness moderated treatment response, with those high in private self-consciousness (i.e., individuals who engaged in more thinking and discussing matters of the self) exhibiting poorer maintenance outcomes when randomized to the automated advisor compared to the human advisor. To the best of the authors’ knowledge, this is the first set of analyses suggesting that baseline psychosocial factors can moderate initial and longer-term efficacy of two interventions with similar content and contact time, but delivered with different communication modalities.

Our current research suggests that if individuals have low amotivation (i.e., higher initial interest in being active) at baseline, an automated advisor may actually be preferable over a human advisor. In addition, the automated advisor has the potential to be more cost-effective and less restrictive for wide-scale adoption, adding to its potential utility [13]. Our results further suggest, however, that a human advisor may be required if someone has higher baseline levels of physical activity amotivation (i.e., less initial interest in being active), given its association with 12-month behavioral initiation, or higher baseline private self-consciousness (given its association with 18-month behavioral maintenance).

Future research is indicated to better explain these results using appropriately designed research studies with *a priori* hypotheses. One potential hypothesis to explain these results could be that even though human and computer advisors utilized the same content, the human advisors may have provided subtle but important customized support and feedback to amotivated participants that were not provided by the automated advisor which proved valuable for counteracting this initial lack of interest. This hypothesis could be explored further by conducting content analysis of human advisor-participant interactions (see: [35] for methods). Such information, while not available for all counseling interactions in the current study, could potentially help to inform further development of automated system interactions [36]. If these subtle differences can be found and linked with improved participant response, they could conceivably be better modeled into automated systems such as via improvements to the overall scripts and logic for amotivated individuals or doing work akin to Timothy Bickmore’s work [37], which focuses on better building in subtle interaction styles within technology-based health promotion systems. In addition, this hypothesis implicitly acknowledges that amotivation might decrease more over time with the human advisor compared to the computer. Post hoc exploration did indeed find a trend for this, whereby amotivation decreased in the human advisor arm and remained steady in the automated advisor arm, but this trend was not significant. As such, this point on the stability of amotivation over time requires additional empirical evaluation.

Interestingly, different psychosocial factors were key moderators for maintenance of physical activity. Specifically, individuals high in private self-consciousness (i.e., individuals who are more inwardly focused) had less favorable maintenance effects if they were matched with an automated advisor. Future research should explore possible explanations for these results using *a priori* hypotheses. For example, one hypothesis for explaining such results is that individuals who are more immersed in issues of the self may require more attentive responses from the advisor to aid in longer-term physical activity maintenance. These results are in line with other results from the CHAT trial reported elsewhere [17] that more individuals in the human arm voluntarily called in during the 18-month follow-up period than those in the automated advice arm. Such increased advisor contact during the maintenance phase could be a potentially useful tool for those high in private self-consciousness. This hypothesis suggests that private self-consciousness is a relatively stable trait-like characteristic, which our own data suggested is true. On average, the standard deviation across time per individual on private self-consciousness was 2.4; for context the possible range of the scale is from 1-26. This stability of private self-consciousness requires further empirical evaluation. It would be worthwhile to explore whether these results could be replicated in other studies focused on maintenance. Similar to the amotivation results, another possibility is that our human advisors provided subtle shifts in the script that proved valuable for those high in private self-consciousness. Based on this, similar exploration of these recorded advisors might prove valuable at identifying these improvements to the script that could then be incorporated into a future automated advisor script/logic.

The trend towards a significant interaction effect between family social support for exercise and the human advisor intervention arm in predicting 18-month physical activity suggests that having higher initial family support may help to enhance the support being received by a human advisor across the longer term. Contrary to expectations, social support did not act as a “buffer” for reduced social contact among those individuals randomized to the automated advice. Given the speculative nature of these observations based on only trend results, replication, particularly with a more stringent empirical evaluation and possibly different measures, is required before any firm conclusions or concrete hypotheses can be drawn.

An alternative but perhaps equally important implication of this work is the fact that the majority of the moderators explored, including all demographics (i.e., age, gender, ethnicity, and marital status), did not moderate the impact of the two interventions. We are cautious about making any strong conclusions from this and call for additional studies to confirm our findings. However, if these future studies replicate our findings then both human and an automated advisors may promote increases in physical activity, regardless of many if not most possible psychosocial moderator variables. As such, the decision between using a human vs. an automated advisor could be based on other issues beyond efficacy such as resource constraints.

### Implications

Overall, the moderator variables explored in this study are reasonably short self-report measures and thus would be relatively easy to administer prior to providing an intervention in real-world settings. This allows for the possibility of an evidence-based, low cost method for identifying who will respond better to an automated advisor or a human advisor for physical activity promotion prior to starting an intervention program. This information, assuming replication of results with other human and automated advice comparisons, has the potential to be valuable for providing empirical evidence to support later clinical decision making and by extension improving the overall efficacy/effectiveness of interventions. Of particular value for cost-effectiveness is the future exploration of combining different intervention delivery media (e.g., human, automated advisor, smartphone, or webpages) to capitalize on the inherent strengths of each media while minimizing their potential weaknesses for physical activity promotion. Further, these results may be interpreted differently in other research communities such as human-computer interaction or communications research, which might place stronger emphasis on understanding and delineating the impact of differences in communication style between humans vs. human-computer interactions [37,38]. As such, further exploration of these questions from a variety of different disciplinary perspectives is required to develop more robust transdisciplinary theories for understanding behavior and behavior change technologies [39,40].

### Limitations

This investigation has several limitations. The study focused on secondary data analyses of a previously conducted trial. As such, the sample was not explicitly powered to detect interaction effects and the overall stability and generalizability of the results are difficult to determine. Despite the potential for reduced power, moderator effects were obtained. However, other theoretically plausible mechanisms such as social anxiety may still moderate treatment efficacy but will need to be explored in a larger sample. This point is also relevant for the trend found for family social support, which needs to be replicated using a larger, more properly powered trial. As suggested by King et al. [9], this type of exploratory analysis is a key first step for generating hypotheses and is conceptually distinct from hypothesis testing endeavors. The results provide guidance for subsequent hypothesis-testing endeavors [9]. Finally, this sample included individuals who signed up to be part of an intervention trial. As such, the amotivation variable would likely be more restricted within this sample than within the more general population. This restricted range may have at least partially contributed to the relatively low reliability estimates within the scale.

## Conclusion

Future research should replicate the study results utilizing appropriate statistical power to confirm the impact of the moderators explored here, including the possibility of null results. In addition, future research should explore experimental studies testing the hypotheses generated by the authors from these secondary analyses (along with counter hypotheses generated by others for explaining the results). Some potential hypotheses to explore include: (a) for individuals who report reduced interest in being active initially, a human advisor provides subtle but important customized support and feedback not provided by the automated advisor, which counteracts this initial lack of motivation; (b) individuals who are interested in discussing matters of the self (i.e., high in private self-consciousness) require the option of contacting a human during the maintenance phase of an intervention; or (c) plausible moderator variables do not in fact moderate the impact of an intervention delivered either by a human or a computer, therefore allowing for resources to drive clinical decisions with regard to their use. In addition, other research could include conducting studies in which participants are differentially assigned (i.e., systematically matched versus mismatched) to a targeted intervention delivery channel based on key moderators (i.e., amotivation, private self-consciousness). Future research also should compare other common and potentially cost-effective treatment models (e.g., print-based media) along with other technologies such as smartphones [14] for the development of strategies for providing appropriately targeted interventions to the individuals who will respond best to them. As suggested by our different results for initiation vs. maintenance, future research should explore which delivery channel should be used at which time during the trial. For example, individuals could start with an initial meeting with a human advisor, transition to an automated advisor, and then, during maintenance switch to a smartphone application. Additional empirical data would be useful to explore these different possibilities and combinations, using empirical design strategies such as a sequential multiple assignment randomized trial (SMART) [41]. Through applying this type of systematic process, advances toward answering the “whiches conundrum” [i.e., identifying which message, for which person, using which delivery-mechanism, within which context, at which time-point [42]] may occur more quickly, resulting in a broadened impact on the public’s health.

## Abbreviations

CHAT: Community health advice by telephone; Mod+: Moderate- to vigorous-intensity physical activity; PAR: Stanford 7-day physical activity recall; TSRQ-E: Treatment self-regulation questionnaire for exercise; SCS-R: Self-consciousness survey-revised; SSE: Social support for exercise; Amot: Amotivation; PSC: Private self-consciousness; SS: Social support.

## Competing interests

The authors declare that they have no competing interests.

## Authors’ contributions

EBH conceptualized the study, ran all statistical analyses, and drafted the manuscript; MPB aided in study design and with statistical analyses and provided feedback on drafts to the manuscript; JO aided in study design and with statistical analyses and provided feedback on drafts to the manuscript; CC, participated in the original intervention including design and implementation of the study and provided feedback on drafts to the manuscript; LG aided with statistical analyses and editing of the manuscript and provided feedback on drafts to the manuscript; BM, participated in the original intervention including design and implementation of the study and provided feedback on drafts to the manuscript; RLM, participated in the original intervention including design and implementation of the study and provided feedback on drafts to the manuscript; MN, participated in the original intervention including design and implementation of the study and provided feedback on drafts to the manuscript; ACK, was the principal investigator on the major trial and therefore participated in the original intervention including design and implementation of the study and provided feedback on drafts to the manuscript; All authors read and approved the final manuscript.

## References

[B1] Center for Disease Control and Prevention Surveillance for certain health behaviors among states and selected local areas—United States, 2010 MMWR Morb Mortal Wkly Rep 2013 61 1 252 23718989

[B2] Marcus BH Williams DM Dubbert PM Sallis JF King AC Yancey AK Franklin BA Buchner D Daniels SR Claytor RP Physical activity intervention studies: what we know and what we need to know: a scientific statement from the American heart association council on nutrition, physical activity, and metabolism (subcommittee on physical activity); council on cardiovascular disease in the young; and the interdisciplinary working group on quality of care and outcomes research Circulation 2006 114 2739 2752 10.1161/CIRCULATIONAHA.106.179683 17145995

[B3] Kahn EB Ramsey LT Brownson RC Heath GW Howze EH Powell KE Stone EJ Rajab MW Corso P The effectiveness of interventions to increase physical activity. A systematic review Am J Prev Med 2002 22 73 107 11985936 10.1016/s0749-3797(02)00434-8

[B4] Kraemer HC Kiernan M Essex M Kupfer DJ How and why criteria defining moderators and mediators differ between the Baron & Kenny and MacArthur approaches Health Psychol 2008 27 S101 S108 18377151 10.1037/0278-6133.27.2(Suppl.).S101 PMC3376898

[B5] Glanz K Bishop D The role of behavioral science theory in development and implementation of public health interventions Annu Rev Public Health 2010 31 399 418 10.1146/annurev.publhealth.012809.103604 20070207

[B6] King AC Marcus B Ahn D Dunn AL Rejeski WJ Sallis JF Coday M Identifying subgroups that succeed or fail with three levels of physical activity intervention: the activity counseling trial Health Psychol 2006 25 336 347 16719605 10.1037/0278-6133.25.3.336

[B7] Michael YL Carlson NE Analysis of individual social-ecological mediators and moderators and their ability to explain effect of a randomized neighborhood walking intervention Int J Behav Nutr Phys Act 2009 6 11 10.1186/1479-5868-6-11 19643024 PMC2728705

[B8] Conn VS Hafdahl AR Moore SM Nielsen PJ Brown LM Meta-analysis of interventions to increase physical activity among cardiac subjects Int J Cardiol 2009 133 307 320 10.1016/j.ijcard.2008.03.052 18582959 PMC2702092

[B9] King AC Ahn DF Atienza AA Kraemer HC Exploring refinements in targeted behavioral medicine intervention to advance public health Ann Behav Med 2008 35 251 260 10.1007/s12160-008-9032-0 18568380 PMC7155916

[B10] King AC Friedman R Marcus B Castro C Napolitano M Alm D Baker L Ongoing physical activity advice by humans versus computers: the community health advice by telephone (CHAT) trial Health Psychol 2007 26 718 727 18020844 10.1037/0278-6133.26.6.718

[B11] Fjeldsoe BS Marshall AL Miller YD Fjeldsoe BS Marshall AL Miller YD Behavior change interventions delivered by mobile telephone short-message service Am J Prev Med 2009 36 165 173 10.1016/j.amepre.2008.09.040 19135907

[B12] Cugelman B Thelwall M Dawes P Online interventions for social marketing health behavior change campaigns: a meta-analysis of psychological architectures and adherence factors J Med Internet Res 2011 9 3 e26 13:84-107 10.2196/jmir.1367 PMC3221338 21320854

[B13] Friedman RH Automated telephone conversations to assess health behavior and deliver behavioral interventions J Med Syst 1998 22 2 95 102 22:95-102 10.1023/A:1022695119046 9571516

[B14] King AC Hekler EB Grieco LA Winter SJ Sheats JL Buman MP Banerjee B Robinson TN Cirimele J Harnessing different motivational frames via mobile phones to promote daily physical activity and reduce sedentary behavior in aging adults PLoS One 2013 8 e62613 10.1371/journal.pone.0062613 23638127 PMC3636222

[B15] King AC Friedman R Marcus B Castro C Forsyth L Napolitano M Pinto B Harnessing motivational forces in the promotion of physical activity: the community health advice by telephone (CHAT) project Health Educ Res 2002 17 627 636 10.1093/her/17.5.627 12408207

[B16] Physical Activity Guidelines Advisory Committee Physical Activity Guidelines Advisory Committee Report, 2008 2008 Washignton, DC: U.S. Department of Health and Human Services

[B17] King AC Hekler EB Castro CM Buman MP Marcus BH Friedman RM Napolitano MA Exercise advice by humans versus computers: maintenance effects at 18 months Health Psycholin press 10.1037/a0030646 PMC7140764 23421896

[B18] Rothman AJ Toward a theory-based analysis of behavioral maintenance Health Psychol 2000 19 64 69 10709949 10.1037/0278-6133.19.suppl1.64

[B19] Noar SM Benac CN Harris MS Does tailoring matter? meta-analytic review of tailored print health behavior change interventions Psychol Bull 2007 133 673 693 17592961 10.1037/0033-2909.133.4.673

[B20] Deci EL Ryan RM Intrinsic motivation and self-determination in human behavior 1985 New York: Plenum

[B21] Pinto B Friedman R Marcus BH Kelley H Tennstedt S Gillman MW Effects of a computer-based, telephone-counseling system on physical activity Am J Prev Med 2002 23 2 113 120 10.1016/S0749-3797(02)00441-5 12121799

[B22] Jarvis KL Friedman RH Heeren T Cullinane PM Older women and physical activity: using the telephone to walk Wom Healt Iss 1997 7 24 29 10.1016/S1049-3867(96)00050-3 9009864

[B23] Blair SN Haskell WL Ho P Paffenbarger RS Vranizan KM Farquhar JW Wood PD Assessment of habitual physical activity by seven-day recall in a community survey and controlled experiments Am J Epidemiol 1985 122 794 804 3876763 10.1093/oxfordjournals.aje.a114163

[B24] Sallis JF Haskell WL Wood PD Physical activity assessment methodology in the five-city project Am J Epidemiol 1985 121 91 106 3964995 10.1093/oxfordjournals.aje.a113987

[B25] Gross LD Sallis JF Buono MJ Roby JJ Nelson JA Reliability of interviewers using the seven-day physical activity recall Res Q Exerc Sport 1990 61 321 325 10.1080/02701367.1990.10607494 2132889

[B26] Dubbert PM Vander Weg MW Kirchner KA Shaw B Evaluation of the 7-day physical activity recall in urban and rural men Med Sci Sports Exerc 2004 36 1646 1654 10.1249/01.MSS.0000139893.65189.F2 15354050

[B27] Levesque CS Williams GC Elliot D Pickering ML Bodenhamer B Finley PJ Validating the theoretical structure of hte treatment self-regulation questionnaire across three different health behaviors Health Educ Res 2007 22 691 702 17138613 10.1093/her/cyl148

[B28] Scheier MF Carver CS The self-consciousness scale: a revised version for use with general populations J Appl Soc Psychol 1985 15 687 699 10.1111/j.1559-1816.1985.tb02268.x

[B29] Sallis JF Grossman RM Pinski RB Patterson TL Nader PR The development of scales to measure social support for diet and exercise behaviors Prev Med 1987 16 825 836 10.1016/0091-7435(87)90022-3 3432232

[B30] Resnick B Orwig D Magaziner J Wynne C The effect of social support on exercise behavior in older adults Clin Nurs Res 2002 11 1 10 21 11:52-70 11845515 10.1177/105477380201100105

[B31] SAS Institute Inc SAS 9.2 TS Level 2M2 2008 Cary, NC USA: SAS Institute Inc

[B32] Aiken LS West SG Multiple regression: testing and interpretting interactions 1991 Newbury Park, London, UK: Sage

[B33] Cronbach LJ Furby L How we should measure “Change” or should we? Psychol Bull 1970 74 68 80

[B34] Simkin-Silverman LR Wing RR Boraz MA Kuller LH Simkin-Silverman LR Wing RR Boraz MA Kuller LH Lifestyle intervention can prevent weight gain during menopause: results from a 5-year randomized clinical trial Ann Behav Med 2003 26 212 220 10.1207/S15324796ABM2603_06 14644697

[B35] Moyers TB Miller WR Hendrickson SML How does motivational interviewing work? therapist interpersonal skill predicts client involvement within motivational interviewing sessions J Consult Clin Psychol 2005 73 590 598 16173846 10.1037/0022-006X.73.4.590

[B36] King AC Bickmore TW Campero I Pruitt L Yin LX Employing ‘virtual advisors’ in prevention care for underserved communities: results from the COMPASS study J Health Communin press 10.1080/10810730.2013.798374 PMC7187757 23941610

[B37] Bickmore T Schulman D A virtual laboratory for studying long-term relationships between humans and virtual agents Proceedings of the 8th International Conference on Autonomous Agents and Multi-agent Systems (AAMAS ’09) 2009 1 297 304

[B38] Nass C Moon Y Machines and mindlessness: social responses to computers J Soc Issues 2000 56 81 103 10.1111/0022-4537.00153

[B39] Hekler EB Klasnja P Froehlich JE Buman MP Mind the theoretical gap: interpreting, using, and developing behavioral theory in HCI research Proceedings of the SIGCHI Conference on Human Factors in Computing Systems 2013 3307 3316

[B40] Riley WT Rivera DE Atienza AA Nilsen W Allison SM Mermelstein R Health behavior models in the age of mobile interventions: are our theories up to the task? Transl Behav Med 2011 1 53 71 10.1007/s13142-011-0021-7 21796270 PMC3142960

[B41] Collins LM Murphy SA Strecher V The multiphase optimization strategy (MOST) and the sequential multiple assignment randomized trial (SMART): new methods for more potent eHealth interventions Am J Prev Med 2007 32 S112 S118 10.1016/j.amepre.2007.01.022 17466815 PMC2062525

[B42] King AC Stokols D Talen E Brassington GS Killingsworth R Theoretical approaches to the promotion of physical activity: forging a transdisciplinary paradigm Am J Prev Med 2002 23 15 25 10.1016/S0749-3797(02)00470-1 12133734

